# Joint modelling of longitudinal 3MS scores and the risk of mortality among cognitively impaired individuals

**DOI:** 10.1371/journal.pone.0182873

**Published:** 2017-08-16

**Authors:** Chris B. Guure, Noor Akma Ibrahim, Mohd Bakri Adam, Salmiah Md Said

**Affiliations:** 1 Department of Biostatistics, School of Public Health, University of Ghana, Accra, Ghana; 2 Institute for Mathematical Research, Universiti Putra Malaysia, Serdang, Malaysia; 3 Department of Mathematics, Faculty of Science, Universiti Putra Malaysia, Serdang, Malaysia; 4 Department of Community Health, Faculty of Medicine & Health Sciences, Universiti Putra Malaysia, Serdang, Selangor, Malaysia; Nathan S Kline Institute, UNITED STATES

## Abstract

**Background:**

Modified Mini-Mental State Examination (3MS) is an instrument administered by trained personnel to examine levels of participants’ cognitive function. However, the association between changes in scores over time and the risk of death (mortality) is not known. The aims of this study are to examine the association between 3MS scores and mortality via cognitive impairment among older women and to determine individuals’ risk of changes in scores to better predict their survival and mortality rates.

**Methods:**

We propose a Bayesian joint modelling approach to determine mortality due to cognitive impairment via repeated measures of 3MS scores trajectories over a 21-year follow-up period. Data for this study are taken from the Osteoporotic Fracture longitudinal study among women aged 65+ which started in 1986–88.

**Results:**

The standard relative risk model from the analyses with a baseline 3MS score after adjusting for all the significant covariates demonstrates that, every unit decrease in a 3MS score corresponds to a non-significant 1.059 increase risk of mortality with a 95% CI of (0.981, 1.143), while the extended model results in a significant 0.09% increased risk in mortality. The joint modelling approach found a strong association between the 3MS scores and the risk of mortality, such that, every unit decrease in 3MS scores results in a 1.135 (13%) increased risk of death via cognitive impairment with a 95% CI of (1.056, 1.215).

**Conclusion:**

It has been demonstrated that a decrease in 3MS results has a significant increase risk of mortality due to cognitive impairment via joint modelling, but insignificant when considered under the standard relative risk approach.

## Introduction

Joint modelling of longitudinal and time-to-event data has witnessed an explosion in the literature of recent years by a good number of researches. A joint modelling approach is used when one is interested in modelling the association between an endogenous variable measured repeatedly over a time period with the interest of determining the risk of an event occurring. De and Tu [1], proposed the longitudinal sub-model to model the progression of a CD4-lymphocyte count with some level of measurement errors and its association with other risk factors via the survival sub-model. De and Tu [1], implemented their approach using the random effects model which enabled them describe the progression in the CD4 cell counts and the effects of the CD4 trajectory on survival of patients. Tsiatis et al. [2], also proposed a two-stage approach in modelling CD4 counts, which according to the authors was a potential marker for human immune virus (HIV) trials, due to its observed correlation with clinical outcomes. The Cox proportional hazards regression model can be used to fit time-dependent covariates, but the CD4 counts are always measured periodically and with substantial errors in their measurements making it impossible for the Cox model to be used, [2]. They therefore modelled the CD4 counts via a repeated random effects model while the other variables were considered under the relative risk (Cox proportional hazards) model.

A study based on the joint longitudinal-survival-cure rate models were proposed and used to analyse data emanating from prostate cancer patients treated with radiation therapy, [3]. They modelled the cured-fraction via a logistic function with the baseline covariates, a time-dependent proportional hazards model for the susceptible group and a non-linear hierarchical mixed model for PSA data. Other similar works include, Proust and Taylor [4], Taylor et al. [5] and Yu et al. [6].

Research in cystic fibrosis is mostly studied to observe the impact of patients’ lung trajectory on the risk of acquiring different types of infections and how the variability of the different lung function quantiles are distributed. Due to the measurement errors introduced as a result of repeated observations, Waldmann and David [7] proposed and applied the Bayesian joint models and came to the conclusion that taking into account the heteroscedastic structure via the longitudinal sub-model helps predict the risk of the disease more accurately.

Other studies that explored the joint modelling approach in different fields of study include, [8, 9]. Andrinopoulou et al. [8], looked at the valve function of cardiac-thoracic surgery which is monitored over a period of time. The joint modelling approach was the most appropriate in helping a physician scan for the trend in valve functions so that they were able to plan their next intervention, [8]. Their approach was implemented via P-splines using Bayesian methods of joint modelling that enabled them specify a time-varying coefficient to link the longitudinal and the survival processes.

The main focus of this work is to examine the modified Mini-Mental State Examination (3MS) which is considered one of the most important tools for the preliminary diagnosis of cognitive functioning. It is a standard instrument used all over the world with some modifications for country specific surveys. This instrument is administered to participants during every visit in a survey by trained personnel to enable medics have a first-hand diagnosis of their patients’ level of cognition. We used dataset obtained from the Osteoporotic Fracture Study conducted over a 21-year follow-up where 3MS scores were recorded repeatedly (at every visit), except visits 3 and 4, resulting in the measurements taking for 7 out of the 9 visits.

The objectives of this study are: 1) To examine and assess the association between 3MS and the risk of dying through cognitive impairment. 2) To propose an appropriate statistical model suitable for examining the behaviour of the longitudinal measures of 3MS over time while modelling the risk of death among cognitively impaired individuals. 3) To calculate an individual’s predictive survival probability and calculate longitudinal trajectories via a shared-parameter modelling approach.

### What is known

It is established in literature that the best way to use the modified Mini-Mental State Examination (3MS) scores to determine its effect on cognitive decline is to consider a two time point (visits) measurement with the assumption that a difference of at least three between these measures contributes significantly to an individual’s cognitive status.

### What this study adds

This is the first ever study to;

- consider a longitudinal measurements of modified Mini-Mental State Examination over a 21-year follow-up dataset which is considered to be measured with errors,
- link the 3MS variable directly to mortality of individuals via cognitive impairment,
- jointly model the relationship between the repeated measures of variable 3MS and the mortality risk using standard and extended relative risk, and linear mixed effects models,
- establish the possibility of predicting participants’ survival probability based on their 3MS scores to enable physicians have a better understanding of their patients’ risk of mortality via cognitive impairment,
- propose a model that is capable of accurately predicting future 3MS scores based on their previous measurement(s) to enable practitioners keep track of whether their patients’ health is deteriorating or not.

## Methods

### Data source and description

Data for our study were acquired from the Study of Osteoporotic Fractures (SOF), [10]. Details of the study have been given elsewhere [11, 12], but briefly, SOF is a longitudinal cohort study that comprises 9704 participants of community-dwelling people ≥ 65 years and mostly of white women. All subjects or participants were interviewed and examined at the start of the study in 1986–88. Participants underwent biennial clinic visits and completed annual questionnaires, [11]. The study was conducted in four sites in the United States, these include, Baltimore, MD; Minneapolis, MN; Monongahela Valley, PA; and Portland, OR. Criteria for inclusion of subjects at the baseline were based on their ability to ambulate without any assistance and with no bilateral hip replacement. Follow-up was carried out for 9 different number of visits (9 different time points) with approximately 2 to 4 years interval between visits.

#### Ethical approval

The SOF study protocol was approved by the Institutional Review Boards at each of the participating clinical sites (University of Maryland, Baltimore; University of Minnesota; University of Pittsburgh; Kaiser Permanente Northwest) and written informed consent was obtained from all participating women prior to enrolment.

#### Data availability

The data used in this manuscript is a secondary data and is publicly available for researchers who wish to make use of it by registering at https://sofonline.epi-ucsf.org/interface/UserLogonFunction.asp?RedirectToURL=/interface/DataDoc.asp. We would like to emphasize that we do not have special access privileges to these data and that interested researchers may apply to access the data as stipulated above.

#### All-cause mortality

Follow-up data on death of participants were obtained from four clinics which individually screened and collected SOF mortality data. In adjudicating all deaths among the SOF subjects along with their other medical records, a State Registered Certificate of Death submitted information to the SOF Coordinating Centre. Before pre- and final adjudication of diagnostic of causes of death among the participants, the Principal Investigator at each clinic site indicated his/her initial diagnoses of death. The causes of death were determined according to the International Classification of Diseases, 9th Revision Clinical Modification (ICD-9-CM). The Endpoint Specialist at the Coordinating Centre then classified each of these deaths into the following categories: Atherosclerosis, Ischemic Heart Disease, Stroke, Cancer, Breast Cancer, Female Cancer, Colon Cancer, Pulmonary, Cognitive impairment and all Causes (deaths excluding those mentioned above).

#### Measure of cognitive function

A modified Mini-Mental State Examination (3MS) test instrument was administered by trained personnel at all the nine (9) follow-ups, except visits two and three. The 3MS test instrument is a global cognitive functioning test that is designed with concentration, language and memory components to screen for cognitive impairment. The 3MS test ranges from 0 to 26, where the highest score indicates the absence of cognitive impairment. Cognitive impairment relates to a person who shows evidence of cognitive decline with preserved impaired activities of daily living. The most common diagnostic tool that is used to classify people of being either impaired or not is the Mini-Mental State Examination as well as the modified Mini-Mental State Examination. It is well established that 3MS score of less than 23 is a pre-condition for a person to be suspected of not being cognitively sound, [11, 13]. Though 3MS is mostly measured repeatedly over a two or more time points, researchers have always categorized individuals as being cognitively impaired if their current scores are at least 3 points less than their previous scores [11, 13]. In order to make use of the repeated scores taken over a period of time for each individual, we took into consideration the 3MS scores for the entire duration of the study (21 years).

### Covariates

Baseline socio-demographic variables which are potentially related to mortality and were obtained, include age (in years), educational level and marital status (married, single, divorced, widower). Age and education were added to the analysis as continuous variables. Medical history of participants, those ever told by healthcare providers of their medical conditions include: angina, myocardial infarction, diabetes and dementia. These variables were categorised in the study as either “yes”or “no”. Depressive symptoms of subjects were measured using the (GDS-15) scale. According to [14] a GDS-15 score of ≥6 indicates the presence of depression among the individual whiles those <6 indicates absence of depression. Body mass index of participants was also obtained from the measurements of participants’ height and weight, as well as hypertension from both systolic and diastolic blood pressures and categorised according to World Health Organisation definition [15]. Others included smoke status which was classified in terms of never, past and current smokers. Participants were also asked to rate and compare their current state of health with that of their counterparts (similar in age) and categorised as poor, good and excellent. Physical activity of participants was obtained through self-administered questionnaires by first responding to either “yes”or “no”the question, “at least once a week, do you engage in any regular activity (brisk walking, jogging, bicycling, and others) long enough to work up a sweat?”, only those who responded yes were further asked, “how many times do you engage in these per week?”. The physical activity variable was categorised into active and inactive groups (i.e., those who were engaged in at least ones a week exercising were grouped as active and the rest as inactive) for the purpose of this analysis.

### Statistical analysis

All statistical analyses were performed using Stata v.14 (College Station, TX: StataCorp LP) and R version 3.3.2 (R Foundation for Statistical Computing, Vienna, Austria). All descriptive analyses were obtained as means, standard deviations and percentages. Simple Bayesian relative risk models were explored to obtain significant variables for the multivariate analysis. Differences in the baseline characteristics for the continuous variables such as body mass index, education and age were tested using a Bayesian analogy to the two sample t-test distribution(Bayesian Estimation Supersedes the t-Test), [16].

#### Joint modelling framework

The objective of this work is to evaluate the effect or association of 3MS profiles over a period of time on the hazards of death via cognitive impairment. Our proposed modelling framework involves fitting the linear mixed effects’ submodel on the repeated measures of the longitudinal covariate and a survival submodel on the hazards of the event of interest (mortality). These will then be fitted jointly by incorporating the two submodels.

**Fig 1:**
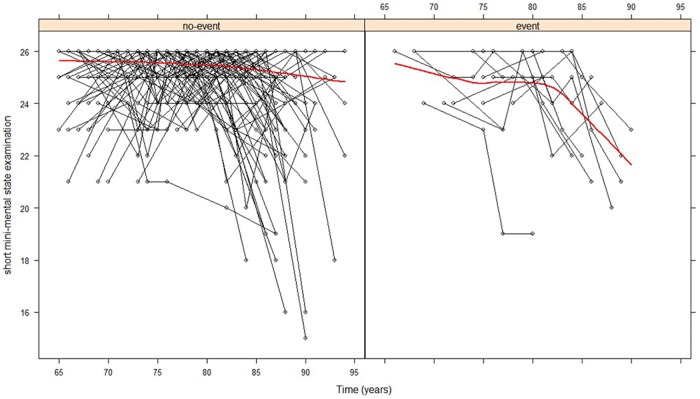
Individual longitudinal trajectories for 100 participants. The left figure represents participants who did not experience the event (mortality due to cognitive impairment) and right are those who experienced the event. The red solid lines denote the observed average longitudinal trajectories.

In order to demonstrate the importance of the joint modelling approach for our study, we present in Fig 1, the longitudinal or individual trajectories for each participant’s 3MS scores. The figure has two sides, to the left are censored subjects and to the right are those who experienced the event (mortality). These graphs are a representation of 100 subjects out of the 1,382 included in our analysis. The graph suggests that at each time point where measurements were taken, the individual participant had his own evolution different from the previous measurement. The individual evolutions further suggest a non-constant score or measure from one time point to another with subject specific intercepts and slopes. It is intriguing to note that the scores for each subject are only known at the time of measurement, implying the risk of survival is only known at that time point. To determine the hazards of death via survival models, these subject-specific intercepts and slopes cannot be modelled directly using survival models. This calls for a shared-parameter modelling approach to uniquely handle data of this kind, the purpose of this study.

The actual unobserved value of the underlying longitudinal trajectories (bio-marker) can be used to quantify the risk or hazard of the covariate to that of the risk of the event. To do this, there is the need to estimate the observed longitudinal bio-marker and then successfully reconstruct the complete longitudinal process or history by using the available measurements for each subject. This is achieved via a linear mixed effects model for the subject-specific evolution over time.

#### Standard and extended relative risk model

The modified Mini-Mental State Examination is measured in different time points, as a result, the standard Cox proportional hazards or relative risk model is not appropriate to be used to model this type of variables. This is so because the proportional hazards assumption is violated. The proportional hazards assumption stipulates that variables must not change over a time period, that is, when measured at the start of the study must stay same throughout. This assumption is violated with time dependent variables such as the 3MS.

If time-varying or dependent covariates are of interest, then the extended relative risk or Cox model may be suitable. However, the extended relative risk model expects the variables to be predictable in nature, for example, seasonal pattern, treatment strategies which may be adjusted during the study period according to some predetermined criterion. Furthermore, the extended relative risk model requires the researcher to have a complete knowledge of the covariate history for all individuals while on study in order to maximize the partial likelihood and thereby estimate the model parameters efficiently and with a higher degree of accuracy. In order to implement this approach, we must understand that the covariate value need to be a time-continuous process measured without error [17]. These types of variables are referred to as exogenous time-varying covariates. The other type known as endogenous covariates are also predictors that can not be predetermined at baseline. An example is a bio-marker where the only way its value can be known is by measurement, for instance CD4 cell count, serum bilirubin levels and mini-mental state examination (MMSE). In order to incorporate these types of covariates into the relative risk model the error terms need to be taken care off via a different methodological approach. The hazards of an event will depend on the actual but unobserved values of the repeated measured variable (bio-marker) at some time point. It is important to quantify the effect of the bio-marker on the hazards or the risk of the event [18].

The relative risk model which is used to model time-to-event outcomes has a baseline function which according to Cox and Hinkley [19] and Anderson and Gill [20] should be left unspecified. Hsieh et al [21] and Rizopoulos et al [22] have indicated that leaving the hazards unspecified may result in an underestimation of the standard errors of the parameter estimates, hence it was estimated using a piecewise constant following Rizopoulos [23].

#### Dynamic predictions of disease-free survival

There is an increasing interest by both practitioners and researchers towards making decisions based on characteristics of subject-specific evolutions for proper medical treatment or care. Personalised medicine helps patients to vary their lifestyle behaviours to mitigate health problems they may encounter later. Joint models provide us with a very useful tool that enables us to determine or predict future survival probabilities at individual levels using subject-specific longitudinal measurements obtained over a period of time [24–26].

If these repeated measurements are obtained up to some known time, then the implication is, that individual did not experience the event of interest up to that time point. This enables us to obtain the conditional subject-specific predictions up to the known time point with the probability that a subject will survive beyond that time, given that the subject has survived up to that known time. Details on how to obtained the posterior predictive distributions for both the survival and longitudinal processes are given elsewhere, Taylor et al. [24], Rizopoulos [26], Rizopoulos [27].

Since these models are implemented via the Bayesian statistical methodology, we have to assign prior distributions to all the joint model parameters so that inferences can be obtain via the posterior distribution. One of the aims of this study is to obtain objective posterior estimates: as a result, non-informative or vague priors were chosen for all parameters. Non-informative normal priors with mean zero (0) and variance 10^5^ were assigned on the coefficients of the relative risk submodel as well as the association parameter *α*. Vague gamma priors with scale and shape parameters as 0.01 were also chosen for the variances associated with the measurement error and that of the random intercept for the longitudinal submodel.

The JMBayes package in R was used to fit our models [26]. Two Markov Chain Monte Carlo (MCMC) chains of 50,000 iterations were performed with the first 10,000 discarded as burn-in to achieve convergence. The 10,000 iterations were discarded after examining some diagnostics plots such as the trace and density plots as well as Brooks-Gelman-Rubin convergence plots as stipulated by Brooks and Gelman [28]. Following the advice of Link and Eaton [29], thinning of chains was not implemented in our analysis. After convergence was achieved, a further 5,000 iterations were performed before posterior summaries were obtained.

## Results

Baseline descriptive data are presented in Table 1, as means, standard deviations and percentages depending on whether the variable is continuous or categorical. The analysis consists of 1382 individual participants out of which 274 (19.83%) experienced the event of interest (mortality) and 1108 (80.17%) did not experience the event. In order to determine whether variables (continuous) differ significantly from each other, a Bayesian version of the frequentist t-test for mean comparisons, “Bayesian Estimation Supersedes the t-Test (BEST)”was used. The results demonstrates that individuals who had the event (mortality) had a higher overall mean age and educational qualification compared to those who did not experience the event of interest and these differences were statistically significantly different with posterior probabilities of 1.00 (100%) and 0.89 (89%) respectively. Also those who had the event had both lower BMI and 3MS scores compared to those who did not, with approximately 0.99 (99%) posterior probabilities. Apart from the continuous variables, all the other categorical variables that were included in the analysis and summarised in Table 1, were significantly related to mortality at the Bayesian bivariate analysis. The overall means(standard deviations) of age, BMI and education were respectively 68.82(3.42), 26.44(2.55) and 12.94(4.27).

**Table 1:** Baseline demographic characteristics of the study population using proportions in brackets for categorical variables and a Bayesian analogy to the classical t-test (BEST) for continuous variables with their posterior means and standard deviations in brackets.

Item	event n = 274	no-event n = 1108	Diff	% PP	Total n = 1382
Age	71.27(3.99)	67.91(2.45)	3.36(1.54)	100	68.82
BMI	25.57(3.95)	26.43(3.92)	-0.86(0.03)	99	26.44
3MS	25.55(0.70)	26.00(0.01)	-0.45(0.70)	99	25.04
EDUC	12.17(0.94)	12.07(0.58)	0.09(0.36)	89.05	12.94
CVD(%)					
yes	7(0.01)	7(0.01)	-	-	14(1.01)
no	267 (0.19)	1101 (0.80)	-	-	1368(98.99)
SMK(%)					
never	188 (0.14)	751 (0.54)	-	-	939(67.95)
past	70 (0.05)	306 (0.22)	-	-	376(27.21)
current	16 (0.01)	51(0.04)	-	-	67(4.85)
COF(%)					
yes	237 (0.17)	991 (0.72)	-	-	1228(88.86)
no	37 (0.03)	117 (0.08)	-	-	154 (11.14)
EXER(%)					
yes	154 (0.11)	713 (0.52)	-	-	867 (62.74)
no	120 (0.09)	395 (0.29)	-	-	515 (37.26)
HYP(%)					
yes	92 (0.07)	311 (0.23)	-	-	403 (29.16)
no	182 (0.13)	797 (0.57)	-	-	979 (70.84)
MARRY(%)					
married	152 (0.11)	718 (0.52)	-	-	870 (62.95)
widow	87 (0.06)	286 (0.21)	-	-	373 (26.99)
separated	0 (0.00)	4 (0.0)	-	-	4 (0.29)
divorced	48 (0.03)	14 (0.01)	-	-	62 (4.49)
never	21 (0.02)	52 (0.04)	-	-	73 (5.28)
COMP(%)					
poor	42 (0.03)	91 (0.07)	-	-	133 (9.62)
good	137 (0.10)	568 (0.41)	-	-	705 (51.01)
excellent	95 (0.07)	449 (0.32)	-	-	544 (39.36)
ALONE					
yes	327 (0.24)	104 (0.08)	-	-	431 (31.19)
no	781(0.56)	170 (0.12)	-	-	951 (68.81)
DIAB					
yes	29 (0.02)	13 (0.01)	-	-	42 (3.04)
no	1079(0.78)	261 (0.18)	-	-	1340 (96.96)

Diff = Difference in the posterior means, PP = Posterior probability, comp = health comparison with peers, smkpast = past smoker, smkcurrent = current smoker, DIAB = diabetes, EXER = exercise, BMI = body mass index, COF = coffee drinker, HYP = hypertensive, CVD = cadiovascular disease, 3MS = modified mini-mental state examination

### Survival submodel

**Fig 2:**
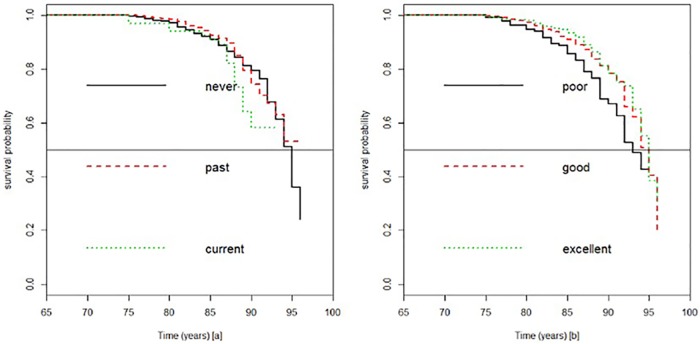
Kaplan-Meier survival probability among individuals smoking status categorised us never, past and current smokers [a] and those who rated their health status compared to age mates as poor, good and excellent[b]. The horizontal line indicates median survival.

In all, there were 1,382 participants’ data included in our analysis. Out of this number, 1108 representing 80.17% did not experience the event, while 274 representing 19.83% experienced the event (mortality) due to cognitive impairment. Provided in Fig 2, are the Kaplan-Meier survival curves obtained according to participants smoking status and a comparison of their individual health against their peers. The median survival rates for excellent and good health condition were (95), each greater than those who rated themselves as with poor condition (93). Those who rated themselves with good and excellent health had approximately equal survival probabilities. Regarding smoking status, subjects who were classified as either past or current smokers had a higher survival compared to those who had never smoked. The standard relative risk model from our analysis assuming a baseline measurement of 3MS scores after adjusting for all the significant covariates, demonstrates that for every unit decrease in a 3MS score, there is a corresponding 1.059 increase risk of mortality with a 95% credible interval of (0.981, 1.143). Except age, bmi, health comparison status and cardiovascular disease, all the other variables did not significantly contribute to predicting mortality under the multivariate Bayesian hazard regression model.

From the results displayed in Table 2, variables such as BMI, COF, EXER and COMP all exhibit a protective effect against people dying via cognitive impairment. It can be said that, for every unit increase in BMI, there is a corresponding decrease in mortality by about 4%. There is a 14% and 22% decrease in mortality among participants who exercise and consume coffee as compared to those who do not though none of them was statistically significant. Cardiovascular disease and type-2 diabetes mellitus are both risk factors of mortality: CVD, but not diabetes is statistically significant, such that the risks among those diagnosed of being DIAB and CVD are respectively 1.68 and 2.73 times those who are not.

**Table 2:** Coefficient and risk ratios of the standard relative risk model to determine the risk of mortality using baseline covariates and with the assumption of a time-independent covariate for 3MS.

	coef	exp(coef)	2.5%	97.5%	se(coef)
compgood	-0.453	0.636	0.448	0.903	0.179
compexcellent	-0.611	0.543	0.374	0.788	0.190
age	0.060	1.062	1.028	1.098	0.017
smkpast	-0.061	0.941	0.711	1.245	0.143
smkcurrent	0.401	1.493	0.886	2.515	0.266
DIAB	0.516	1.675	0.955	2.938	0.287
EXER	-0.153	0.858	0.672	1.096	0.125
BMI	-0.036	0.965	0.936	0.994	0.016
COF	-0.243	0.784	0.551	1.116	0.180
HYP	0.159	1.172	0.906	1.517	0.131
CVD	1.005	2.733	1.270	5.882	0.391
3MS	-0.058	0.944	0.875	1.019	0.039

Coef = Coefficients of the regression model, 95% credibility intervals and exp(Coef) = exponentiated coefficients (risk ratios), comp = health comparison with peers, smkpast = past smoker, smkcurrent = current smoker, DIAB = diabetes, EXER = exercise, BMI = body mass index, COF = coffee drinker, HYP = hypertensive, CVD = cadiovascular disease, 3MS = modified mini-mental state examination

### Joint model

From the results displayed in Table 3, all parameters that were significant under the standard relative risk model were also significant under the joint modelling approach. We observed a significant difference between the estimated error variance given as *σ*_0_ = 1.585 and the estimated random intercept of *σ*_*b*0_ = 2.276, an indication of heterogeneity in the longitudinal measurement of the modified Mini-Mental State Examination that must be accounted for. The extended relative risk model was fitted to the 3MS scores with the assumption that these measurements were taking without errors (there follows a stepwise function). It was observed from the extended relative risk model that the effect of 3MS scores relative to mortality is 0.999, an indication that every unit increase in 3MS scores results in a 0.09% reduced risk of mortality.

**Table 3:** Joint modelling of survival (the standard relative risk model) and longitudinal (linear mixed effects model) data with the effects of baseline covariates and also with the extended relative risk model assuming exogenous time-varying covariate for 3MS.

Variables	Coef	exp(Coef)	2.5%	97.5%
**Relative Risk Submodels**				
**Standard**				
compgood	−0.313	0.732	0.498	1.122
compexcellent	−0.436	0.647	0.461	0.937
age	0.032	1.032	1.009	1.057
smkpast	−0.042	0.959	0.729	1.222
smkcurrent	0.336	1.399	0.844	2.171
DIAB	0.416	1.515	0.791	2.533
EXER	−0.185	0.831	0.641	1.053
BMI	−0.037	0.964	0.941	0.993
COF	−0.255	0.775	0.582	1.076
HYP	0.108	1.114	0.821	1.409
CVD	0.889	2.433	1.110	4.837
**Extended**				
3MS	−0.001	0.999	0.999	0.999
**Joint Models**				
Assoct (*α*)	−0.126	0.881	0.823	0.947
*σ*	1.585		1.559	1.614
*σ*_*b*0_	2.276		0.748	5.086

Coef = Coefficients of the regression model, 95% credibility intervals and exp(Coef) = exponentiated coefficients (risk ratios), comp = health comparison with peers, smkpast = past smoker, smkcurrent = current smoker, DIAB = diabetes, EXER = exercise, BMI = body mass index, COF = coffee drinker, HYP = hypertensive, CVD = cadiovascular disease, Assoct = Association parameter, 3MS = modified mini-mental state examination

The parameter labelled “Assoct”is the parameter that actually measures the association between repeated modified Mini-Mental State Examination and the risk of dying via cognitive decline. Our joint modelling approach found a strong association between the 3MS scores and the risk of mortality, such that, every unit decrease in the 3MS score results in a 1.135 (13%) increased risk of death via cognitive impairment with a 95% credible interval of (1.056, 1.215). These results are statistically significant indicating that indeed 3MS is a good predictor of mortality.

In Table 3, BMI, COF, EXER, and COMP all exhibit a protective effect against people dying via cognitive impairment. For every unit increase in BMI, there is a direct negative effect of mortality by about 4%. There is also a 17% and 23% decrease in mortality among participants who exercise and consume coffee compared to those who do not, though none of these is statistically significant. Cardiovascular disease, hypertension, smoke status and type-2 diabetes mellitus are all risk factors of mortality, none except CVD is statistically significant. Age is a risk factor of mortality due to cognitive impairment, such that, for every unit increase in age, there is a 3% corresponding increase risk of death with a credible interval of (1.009, 1.057).

### Future predictions

**Fig 3:**
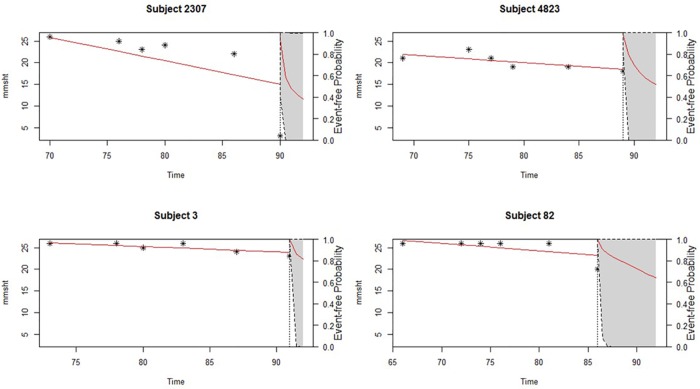
Dynamic predictions of survival probabilities comparing subjects 2307, 4823, 3 and 82 at the end of the entire follow-up with their individual 3MS scores. The asterisks represent individual’s 3MS scores at each visit.

A predictive analysis of individual survival for 4 participants including those who experienced the event and those who did not, were considered based on individual repeated measurements of the 3MS score over the period there were involved in the study. The objective is to obtain the conditional probability of surviving later after having observed their last 3MS measurement. Fig 3 demonstrate two types of predictions that were obtained taking into consideration;

- the 3MS median trajectories are depicted with the solid lines and their corresponding 95% credible intervals
- the conditional survival probabilities are represented by the dotted lines.

**Fig 4:**
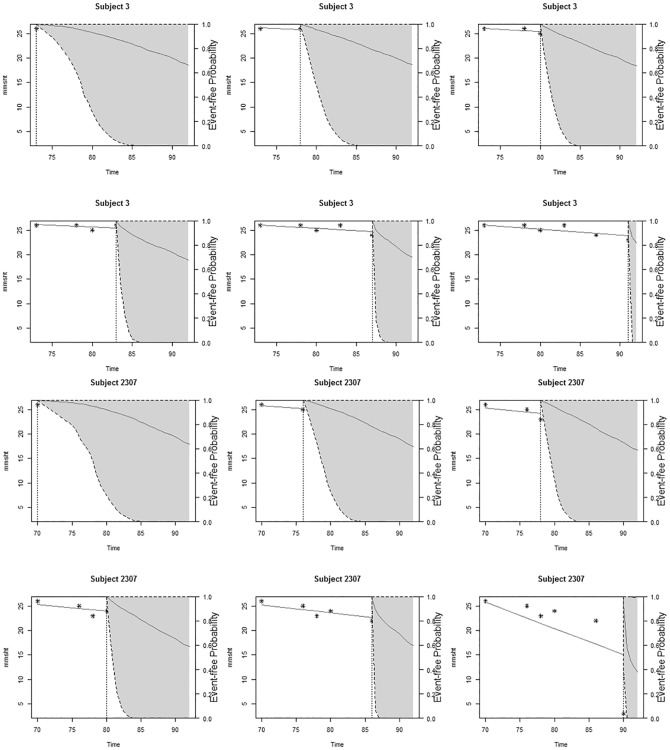
A complete dynamic predictions of survival probabilities comparing subjects 2307 and 3 at each time point for their individual 3MS scores during the entire follow-up period for the purpose of observing these participants survival at every visit with the dotted vertical line indicating the score obtained for that individual at that time point.

Figs 3 and 4, show the evolutions in time of four and two individual participants comprising subjects 2307, 4823, 82 and 3. These participants were randomly chosen for the purpose of a direct comparison among them in order to illustrate their future survival probabilities. As depicted in the figures, the overall predictive survival for subject 2307 is lower than any of the other subjects and this is followed by subject 4823 and then subject 82. Participants with the highest survival among these four is from subject number 3. Fig 3, shows a clear sharp decline of subject 2307’s 3MS score followed by subject 4823, as compared to 3 which appears to be stable. This shows that a decline in 3MS indicates the risk involved in an individual’s survival. This is so because, a decline in 3MS, as showed on the figures, results in a decline in each person’s probability of survival.

To further illustrate our point, Fig 4 shows the complete predictive survival for subjects 2307 and 3. This figure illustrates the ability of the models to predict at each 3MS score an individual’s probability of survival beyond that level having survived up until that time point. Though both at the first time point had a similar survival probability, it is clear from time point 2 and 3 that subject 2307’s survival decreased while subject 3’s survival seemed relatively stable where both can be traced back to each person’s 3MS scores. As subject 3’s predictive survival increases, subject 2307’s continued to decrease. This is an indication that a stable 3MS over a long period is suggestive of higher predictive survival as compared to an unstable or decreased 3MS scores.

**Fig 5:**
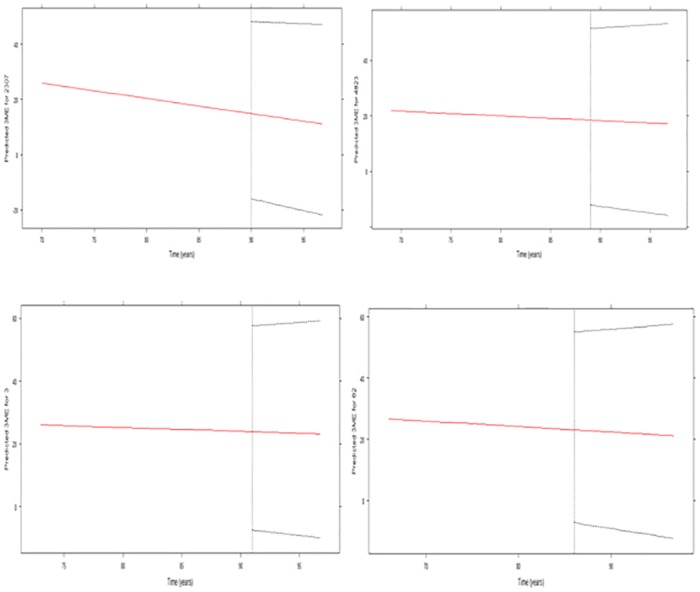
Dynamic predictions of the longitudinal trajectories comparing subjects 2307, 4823, 3 and 82 at the end of the entire follow-up to determine their next 3MS scores. The horizontal red line indicate the current 3MS scores and that of the future predictive scores with the vertical dotted lines indicating the last time the individual was alive.

Apart from the ability to predict survival of individual participants based on their 3MS scores, it has also been demonstrated via Fig 5, which contains the dynamic prediction of an individual’s longitudinal evolutions, that we can obtain or predict the 3MS measurements for each participant which will be of much interest to the physician in addition to the predictions of their survival, since it will help them know before hand their client’s next likely score to recommend or give them better treatment. It is worth mentioning that a look at the predictive survival probability and that of the longitudinal trajectories among the four subjects clearly illustrate participants who show a sharp decline in their future predictive survival and this correspondingly shows a sharp decline in their future predictive modified Mini-Mental State Examination scores, which is an indication that a decrease in 3MS affects an individual’s survival. For instance, subject 2307 shows a faster decline than any of the other subjects in terms of their 3MS, followed by 4823 and then to 82, but remains comparatively stable for subject 3. This is similarly visible in Fig 4, where 3 has the highest predictive survival followed by 82 with 2307 having the lowest.

## Discussion

We have illustrated how joint modelling can be used as an alternative when dealing with repeated measurements and time-to-event data in dynamically predicting an individual’s serial 3MS measurements. Individual mild cognitive impairment diagnosis using modified Mini-Mental State Examination scores may be improved by the use of these types of models which take into consideration or account all the individual scores which have accumulated over a time period.

In this large multi-centred study with a 21-year follow-up of mainly white women above the age of 60, there were statistically significant differences between those who had the event and those who did not in relation to their education, body mass index, modified Mini-Mental State Examination scores and age variables at the univariate level. Though people who died were much older and had higher education compared to others, they had lower body mass index and 3MS scores. The association between education and mortality diminished after accounting or adjusting for other covariates and was dropped from the final model.

In the standard relative risk model, self-assessment of health in comparison with others had a significant relation between good and poor but with the joint modelling approach where 3MS was modelled via linear mixed effects, the association disappeared. It was further observed that the modified Mini-Mental State Examination was an insignificant variable in predicting mortality from cognitive impairment. These findings contradict that of Luck et al. [30], who found a beneficial effect with a Mini-Mental State Examination which differ somehow from the 3MS used in our study. The finding is in consonance with Karp et al. [31] and that of Kerola et al. [32] work, that conclude that MMSE at baseline do not significantly influence mortality. When the scores were taken in totality and assumed to vary with time, but in a step-wise function and modelled via the extended relative risk model with a time-varying covariate approach, it was observed to be a statistically significant predictor of mortality. Though the risk of dying was just 0.09%, the credible interval which includes the true risk ratio was as high as 0.11% and as low as 0.07%.

The longitudinal and survival submodels were both fitted using the linear mixed effects and the relative risk models respectively. Both models were then jointly fitted using an alpha parameter which linked the repeated measurements and the time-to-event variables in predicting mortality via cognitive impairment. The longitudinal submodel contained the fixed effects specified using the age variable while a random effects approach was used for subject specific intercept.

In the joint model approach, 3MS was considered to be measured with errors in which these measurements violate the step-wise function condition of the relative risk model. It became clear that 3MS significantly reduces mortality via cognitive impairment by about 9%. The joint model clearly shows the level of effect one would expect any time there is an increase in the 3MS score, more so than it is with the extended relative risk and also far better than the standard model. These findings are in line with that of Su et al. [33], Cruz et al. [34] and Park et al. [35]. Su et al. [33], revealed that Mini Mental State Examination at baseline can predict mortality by about 23%. Cruz et al. [34] and Park et al. [35] found that a decrease in MMSE is more than two times the risk of predicting mortality among elderly Korean people aged 60 and over. Another findings similar to our is a study by An and Liu [36]. An and Liu [36], categorised the MMSE scores into normal, mild, moderate and severe cognitive impairment and established that as one moves from normal to severe there is a statistically significant increase risk of mortality ranging from 20 to 47 percent among Chinese adults aged 80 and above.

With the dynamic predictive models, the survival and event-free probabilities were calculated for subjects 2307 and 3 taking into account all their modified Mini-Mental State Examination scores that had accumulated over time and had accordingly been updated at each time point where these measurements became readily available. It is believed that this approach could provide very useful evidence-based information that may help the practitioner to individually assess the impact of 3MS on patients’ survival and the best treatment to mitigate mortality via cognitive impairment. The calculated event-free probability can be used as a warning sign to the physician to plan an intervention as early as possible.

One of the most important tools in joint models is its ability to capture or take into consideration the association between the survival time and repeated measurement of a risk factor variable [25]. Though time-dependent relative risk models have been used and are still being used to handle variables that change with time, there is a limitation to which it can be used. A time-dependent relative risk model assumes that the variable that changes with time does so in a step-wise function. This assumption is not appropriate or realistic for certain types of measurements such as 3MS due to the fact that these scores are endogenous (measured with errors) in nature.

In biomedical research where measurements of various outcomes are taking over a time period in an attempt to understand patients health or the risk of an event occurring, the joint modelling approach will be the most useful tool to consider in an effort to link the longitudinal and survival outcomes. Though joint modelling maybe the most suitable approach, it has a low convergence rate mainly because there are a very large number of parameters that need to be estimated when considered under the Markov Chain Monte Carlo method.

Mortality via cognitive impairment or dementia is gradually increasing with the survival rate of individuals diagnosed with dementia, and its subtypes among the elderly stands at 4 to 8 years on the average, [37, 38]. The modified Mini-Mental State Examination is but one of the tools used in diagnosing cognitive impairment, it would therefore be very interesting to include other longitudinal measurements along with the 3MS scores to comprehensively predict the survival probability and longitudinal trajectories of each person.

There are a number of limitations that we need to acknowledge, though the results obtained in this study show an important relationship between the longitudinal measurements of the 3MS trajectories and survival probabilities among American white older women; these findings may not necessarily be generalizable. Efforts were made to evaluate or test for violations, all except the 3MS scores that were included in the model were considered at baseline. Thirdly, as always, the case for prospective studies, there was a high percentage of missing values, which probably would have had an effect on the overall estimate of the true association. There were also a few number of diseases included in this analysis.

Despite the limitations above, there are a good number of strengths in our study. First of all, in almost every study on either morbidity or mortality, of cognitive impairment or dementia, previous research have all considered 3MS at baseline with either the traditional Cox regression or logistic regression model irrespective of the duration and number of measurements recorded. Secondly, our study involved a 21-year cohort which was used to examine the association between 3MS scores trajectories and the risk of death via cognitive impairment or dementia. This study was examined mainly on white older women living in the United states. The United States alone is projected to record about 7.1 million people with dementia by 2025 and 13.8 million by end of 2050, [39]. These findings, we believe will be of much interest to practitioners as well as researchers both within and outside of the study area.

This approach is best used or implemented in a study if interest of researchers on predicting the probability of survival of individual patients over a time period. It can also be used to predict future subject-specific evolutions taking into account their current measurements. It is also best used if a researcher suspects that the variable of interest is measured with errors which violates both the standard and extended relative risk models. In using the joint modelling approach, one is able to account for the unobserved effects (which is significant and as high as 2.276 in our analysis) which affects the accuracy of the effect size estimate.

## Conclusion

Findings from this work has revealed that the risk reduction of death with cognitive impairment or dementia can best be predicted using modified Mini-Mental State Examination scores and analysed via joint models. It has also been demonstrated that a decrease in 3MS results has a significant increase in cognitive impairment mortality. The use of standard and/or extended relative risk or logistic regression models may not be the most appropriate statistical analysis to consider with a variable measured repeatedly over a time period.
